# Bilateral pyosalpinx in a peripubescent female with Hirschsprung's disease: a case report

**DOI:** 10.1186/1865-1380-4-64

**Published:** 2011-10-12

**Authors:** Bobby Desai, Timothy Ward

**Affiliations:** 1 University of Florida, Department of Emergency Medicine, PO Box 100186, Gainesville, FL 32610, USA

## Abstract

This is a case report of bilateral pyosalpinx in a peripubescent female with a history of Hirschsprung's disease. Bilateral pyosalpinx is a rare condition in non-sexually active females. The presence of this disease in a patient with a history of Hirschsprung's disease is concerning for an association of the two processes.

## Introduction

This is a case report of bilateral pyosalpinx in a peripubescent female with a history of Hirschsprung's disease. Bilateral pyosalpinx is a rare condition in non-sexually active females. The presence of this disease in a patient with a history of Hirschsprung's disease is concerning for an association of the two processes.

## Case Report

A 12 year old female presents to the emergency department with a complaint of abdominal pain. She has a past medical history of Hirschsprung's disease with a staged repair. At day four of life she underwent colostomy with resection of the affected colon from the mid transverse colon to the junction of the sigmoid and descending colon. She returned two months later for the second stage of the repair where she underwent a Soave endorectal pull thru procedure with incidental appendectomy. Since that time she has had a good recovery without constipation or diarrhea and with normal bowel function. She had recurrent tonsillitis and underwent tonsillectomy. She takes no medications. The patient complains of a sharp bilateral lower abdominal pain for the past two days that is greatest in the suprapubic region. She has had three episodes of emesis. She denies a change in bowel habits. She does report a low grade fever to 101. She denies dysuria, frequency or hematuria. She denies any history of sexual activity. Her first menstrual period was six weeks ago and her second menstrual period was two weeks ago. She complains of a new watery vaginal discharge for less than one day.

Upon arrival her vitals are temperature 37.2 degrees Celsius by mouth, pulse 125 beats per minute, blood pressure 124/64 mm/Hg, and pulse ox 96% on room air. She weighs 45 kg. She is in obvious moderate distress due to her pain. Her bowel sounds are normal. Her abdomen is non distended and firm with voluntary guarding. It is diffusely tender, but worse in the bilateral lower quadrants without rebound tenderness. There is no CVA tenderness. On pelvic exam, there are normal external genitalia Tanner stage II-III with intact hymen from six o'clock to nine o'clock position. There are no obvious perineal or vaginal lacerations. A watery blood tinged discharge is present. The rest of her physical exam is unremarkable.

Initial labs showed a normal metabolic panel. The complete blood count had a normal hemoglobin and hematocrit with a white blood cell count of 14.5 thou/cu mm. There were 64 percent neutrophils and 18 percent lymphocytes with 11 percent bands. Her urinalysis had 219 red blood cells and 85 white blood cells with a large amount of squamous epithelial cells. It was nitrite negative and had large leukocyte esterase. Urine PCR for gonorrhea and chlamydia was negative. The urine pregnancy test was negative. CT scan of the abdomen and pelvis with IV and oral contrast showed normal lung bases, liver, spleen, pancreas, gallbladder, kidneys, and adrenal glands. There were no bowel obstruction noted. Bilateral dilated tubular structures were noted in the lower quadrants, adnexal regions, with wall enhancement and surrounding inflammatory changes consistent with bilateral pyosalpinx. There were no distinct drainable abscesses seen.

**Figure 1:**
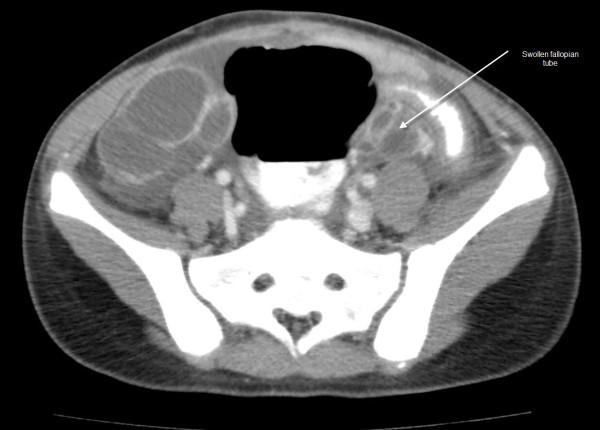
**CT scan of the abdomen and pelvis with intravenous and oral contrast**. Bilateral dilated fallopian tubes with pronounced wall enhancement are shown.

**Figure 2:**
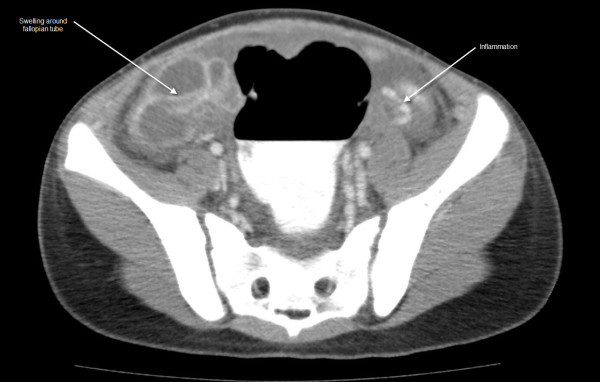
**CT scan of the abdomen and pelvis with intravenous and oral contrast**. Swelling and inflammation are shown around the fallopian tube.

**Figure 3:**
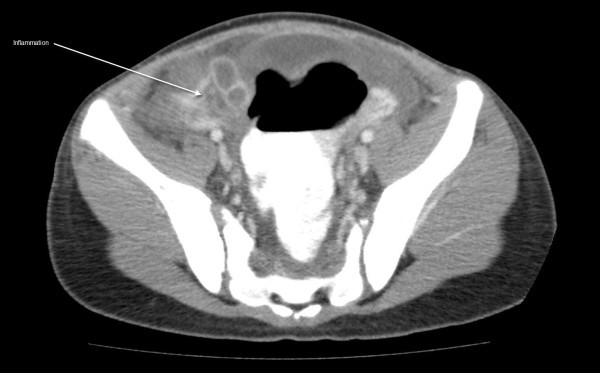
**CT scan of the abdomen and pelvis with intravenous and oral contrast**. Inflammation is demonstrated.

**Figure 4:**
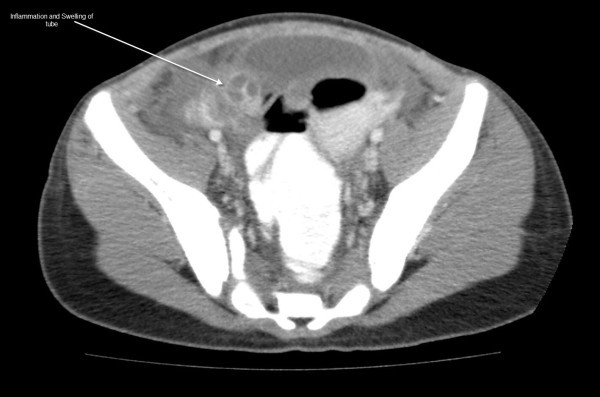
**CT scan of the abdomen and pelvis with intravenous and oral contrast**. A lower cut on the CT scan demonstrating extension of inflammation.

**Figure 5:**
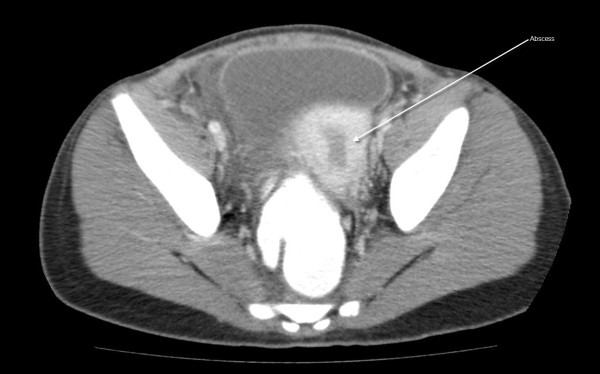
**CT scan of the abdomen and pelvis with intravenous and oral contrast**. Abscess is shown.

See Figures 1, 2, 3, 4 and 5: CT scan of the abdomen and pelvis with intravenous and oral contrast showing bilateral dilated fallopian tubes with pronounced wall enhancement

She received IV fluids and morphine for pain control, and she was admitted to gynecology service for IV antibiotics. In the hospital she received IV Ampicillin, Gentamicin, and Flagyl for four days until she was afebrile for forty eight hours and had a normal white count. She was discharged on a ten day course of Doxycycline and Flagyl with Motrin for pain control. At six month telephone follow up she denies any recurrence of her symptoms.

## Discussion

The presentation of a 12 year old, who is not sexually active, with bilateral pyosalpinx and a history of Hirschsprung's disease is extremely rare. The pathology and anatomical location of these two diseases processes suggest that they may be associated.

Hirschsprung disease is an uncommon congenital disorder that affects 1 in 5000 of live births. The disease is characterized by the absence of ganglion cells in the distal colon including the anus. It results from incomplete migration of neural crest cells or early cell death. There have been eight genes identified that are associated with Hirschsprung disease and the disease has been associated with other congenital abnormalities. Five percent of all cases are associated with Down syndrome [1]. In one prospective study 25 percent of Hirschsprung patients were also found to have congenital abnormalities of the kidney and genital track, the most common abnormalities being hydronephrosis and hypoplasia [2].

The association of hydrosalpinx and Hirschsprung's disease was previously suggested in 2007 by Merlini et al. In this case series the author suggest that bilateral hydrosalpinges is a associated abnormality of Hirschsprung's disease due to neurocristopathy. The paper also discusses the possibility that the disease process could be a complication from the surgical repair. This is the only article that was found in a pubmed search from 1980-present which discusses hydrosalpinx or pyosalpinx in association with Hirschsprung disease [3].

Pyosalpinx is the acute inflammation of the salpinx which is most commonly caused by gonorrhea. Other well described causes of pyosalpinx are Chlamydia and enteric bacteria [1]. Hydrosapinx has been associated with less common organisms including pneumococcus, streptococcus, and shigelloides and is seen in non sexually active females [4-7]. Both pyosalpinx and hydrosalpinx have been reported to present at menarche in females with underlying urogenital malformations [8].

In this case the patient presented just prior to her second menstrual period with pyosalpinx that was confirmed by CT exam. She had complete resolution of symptoms with IV and PO antibiotics and did not have return of symptoms at six month follow up. It is reasonable to speculate that her underlying Hirschsprung's disease attributed to this condition.

## Consent

Written informed consent was obtained from the parents of the patient for publication of this Case report and any accompanying images. A copy of the written consent is available for review by the Editor-in-Chief of this journal.

## Competing interests

The authors declare that they have no competing interests.

## Authors' contributions

TW & BKD: Wrote and edited case report
